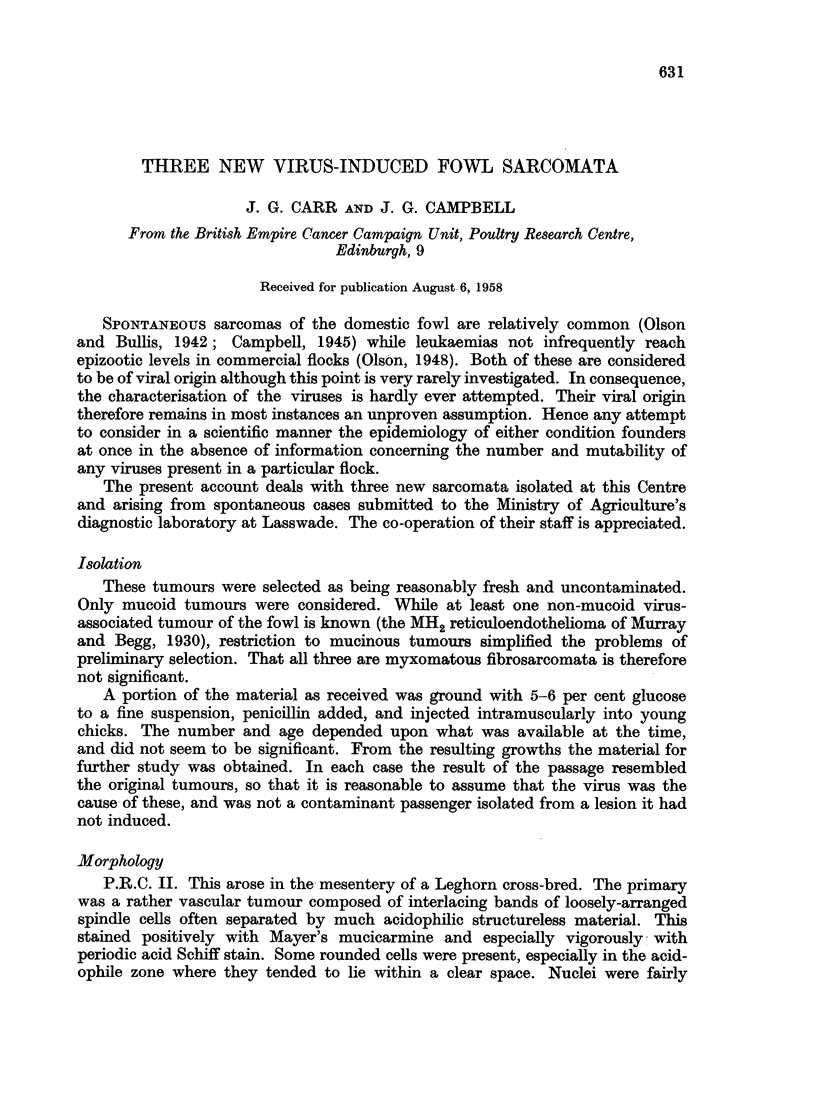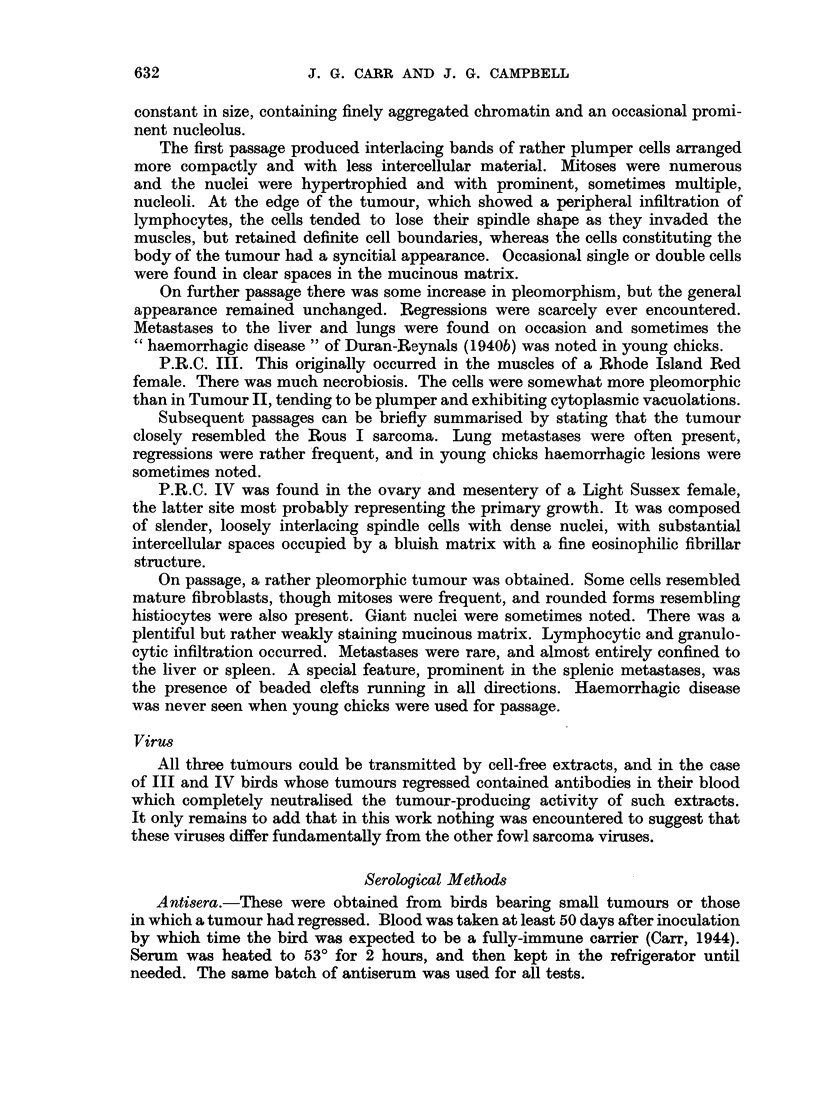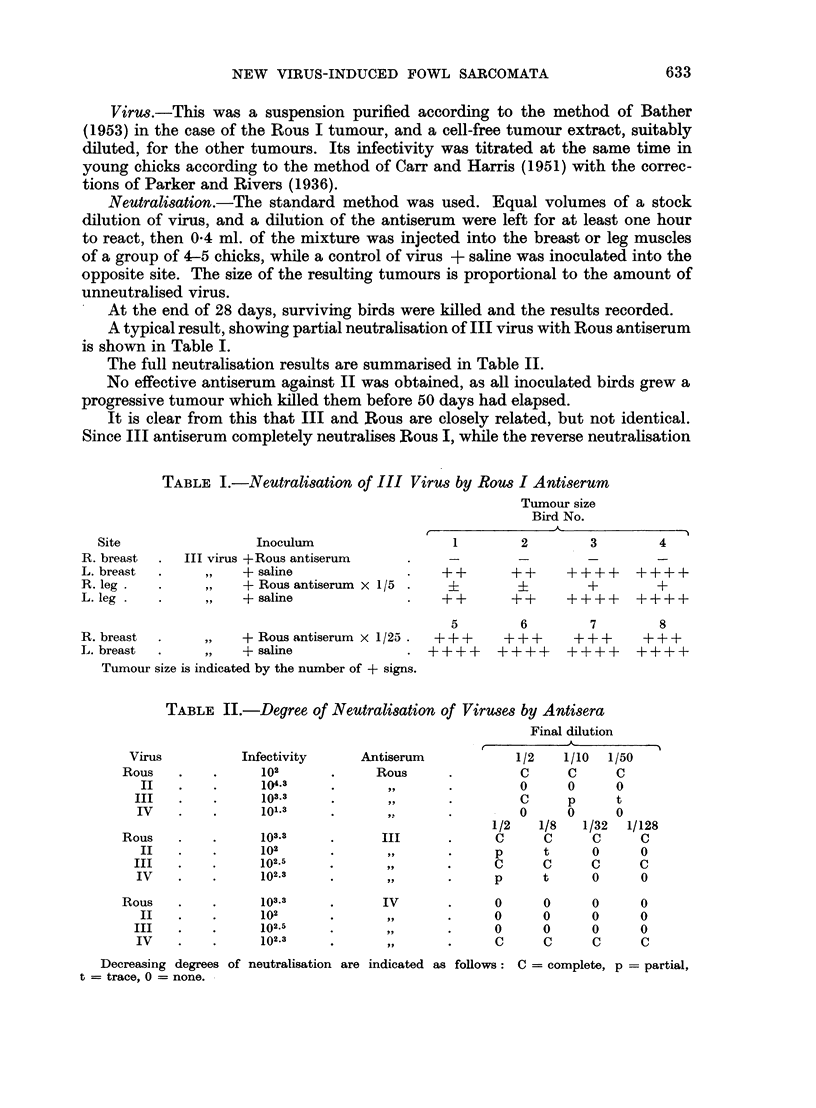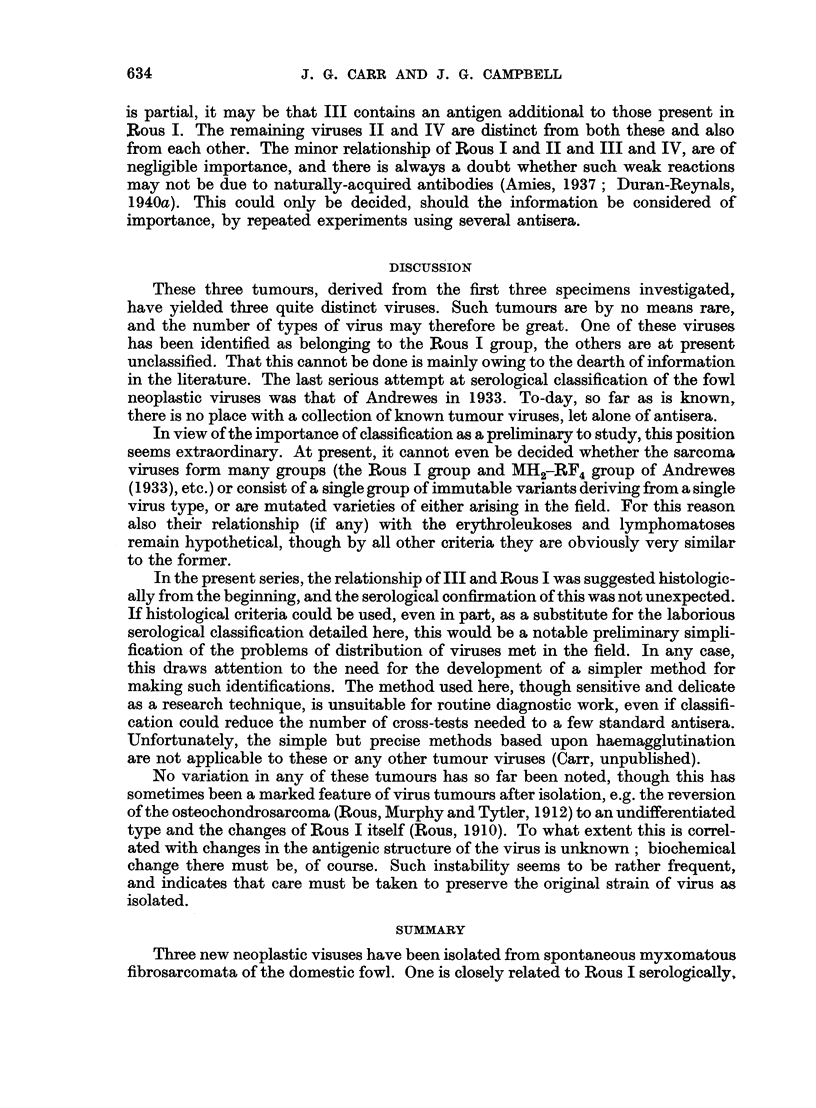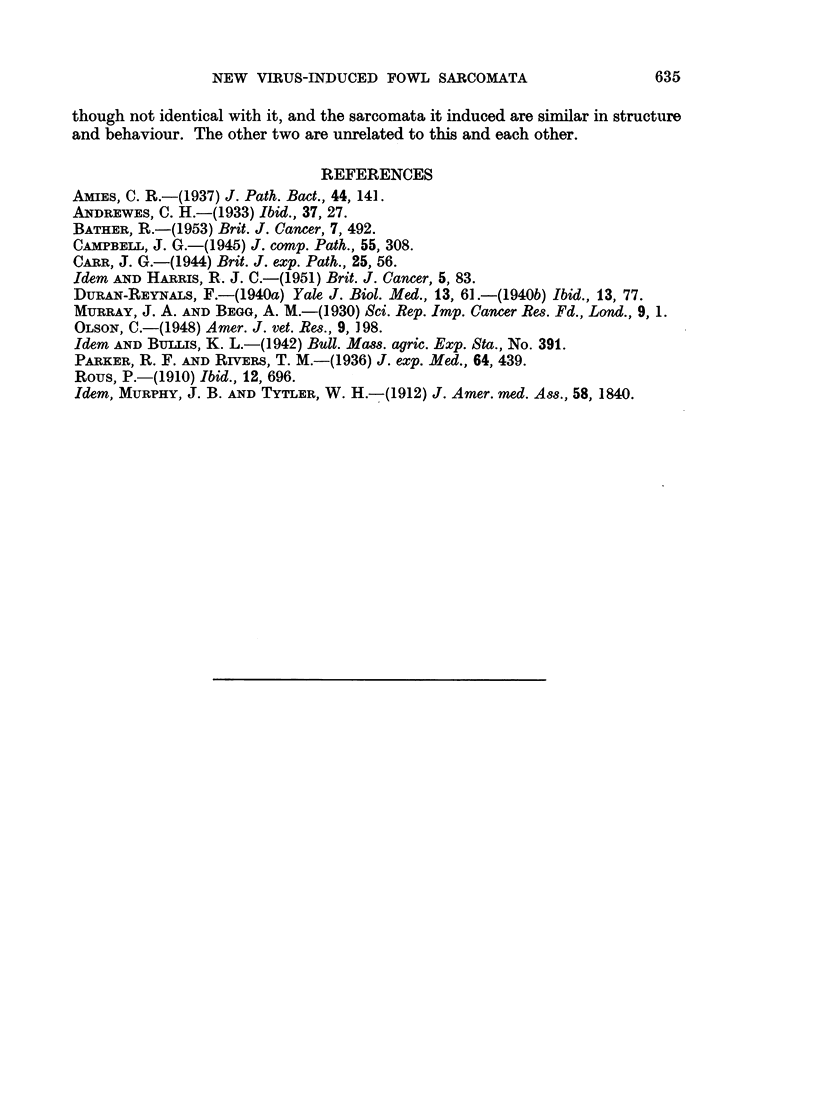# Three New Virus-Induced Fowl Sarcomata

**DOI:** 10.1038/bjc.1958.71

**Published:** 1958-12

**Authors:** J. G. Carr, J. G. Campbell


### THREE NEW VIRUS-INDUCED FOWL SARCOMATA

J. G. CARR AND J. G. CAMPBELL

*From the British Empire Cancer Campaign Unit, Poultry Research Centre, Edinburgh, 9*

Received for publication August 6, 1958

SPONTANEOUS sarcomas of the domestic fowl are relatively common (Olson and Bullis, 1942; Campbell, 1945) while leukaemias not infrequently reach epizootic levels in commercial flocks (Olson, 1948). Both of these are considered to be of viral origin although this point is very rarely investigated. In consequence, the characterisation of the viruses is hardly ever attempted. Their viral origin therefore remains in most instances an unproven assumption. Hence any attempt to consider in a scientific manner the epidemiology of either condition founders at once in the absence of information concerning the number and mutability of any viruses present in a particular flock.

The present account deals with three new sarcomata isolated at this Centre and arising from spontaneous cases submitted to the Ministry of Agriculture's diagnostic laboratory at Lasswade. The co-operation of their staff is appreciated.

*Isolation*

These tumours were selected as being reasonably fresh and uncontaminated. Only mucoid tumours were considered. While at least one non-mucoid virus-associated tumour of the fowl is known (the $MH_2$ reticuloendothelioma of Murray and Begg, 1930), restriction to mucinous tumours simplified the problems of preliminary selection. That all three are myxomatous fibrosarcomata is therefore not significant.

A portion of the material as received was ground with 5–6 per cent glucose to a fine suspension, penicillin added, and injected intramuscularly into young chicks. The number and age depended upon what was available at the time, and did not seem to be significant. From the resulting growths the material for further study was obtained. In each case the result of the passage resembled the original tumours, so that it is reasonable to assume that the virus was the cause of these, and was not a contaminant passenger isolated from a lesion it had not induced.

*Morphology*

P.R.C. II. This arose in the mesentery of a Leghorn cross-bred. The primary was a rather vascular tumour composed of interlacing bands of loosely-arranged spindle cells often separated by much acidophilic structureless material. This stained positively with Mayer's mucicarmine and especially vigorously with periodic acid Schiff stain. Some rounded cells were present, especially in the acidophile zone where they tended to lie within a clear space. Nuclei were fairly constant in size, containing finely aggregated chromatin and an occasional prominent nucleolus.

The first passage produced interlacing bands of rather plumper cells arranged more compactly and with less intercellular material. Mitoses were numerous and the nuclei were hypertrophied and with prominent, sometimes multiple, nucleoli. At the edge of the tumour, which showed a peripheral infiltration of lymphocytes, the cells tended to lose their spindle shape as they invaded the muscles, but retained definite cell boundaries, whereas the cells constituting the body of the tumour had a syncitial appearance. Occasional single or double cells were found in clear spaces in the mucinous matrix.

On further passage there was some increase in pleomorphism, but the general appearance remained unchanged. Regressions were scarcely ever encountered. Metastases to the liver and lungs were found on occasion and sometimes the " haemorrhagic disease " of Duran-Reynals (1940*b*) was noted in young chicks.

P.R.C. III. This originally occurred in the muscles of a Rhode Island Red female. There was much necrobiosis. The cells were somewhat more pleomorphic than in Tumour II, tending to be plumper and exhibiting cytoplasmic vacuolations.

Subsequent passages can be briefly summarised by stating that the tumour closely resembled the Rous I sarcoma. Lung metastases were often present, regressions were rather frequent, and in young chicks haemorrhagic lesions were sometimes noted.

P.R.C. IV was found in the ovary and mesentery of a Light Sussex female, the latter site most probably representing the primary growth. It was composed of slender, loosely interlacing spindle cells with dense nuclei, with substantial intercellular spaces occupied by a bluish matrix with a fine eosinophilic fibrillar structure.

On passage, a rather pleomorphic tumour was obtained. Some cells resembled mature fibroblasts, though mitoses were frequent, and rounded forms resembling histiocytes were also present. Giant nuclei were sometimes noted. There was a plentiful but rather weakly staining mucinous matrix. Lymphocytic and granulocytic infiltration occurred. Metastases were rare, and almost entirely confined to the liver or spleen. A special feature, prominent in the splenic metastases, was the presence of beaded clefts running in all directions. Haemorrhagic disease was never seen when young chicks were used for passage.

### *Virus*

All three tumours could be transmitted by cell-free extracts, and in the case of III and IV birds whose tumours regressed contained antibodies in their blood which completely neutralised the tumour-producing activity of such extracts. It only remains to add that in this work nothing was encountered to suggest that these viruses differ fundamentally from the other fowl sarcoma viruses.

### *Serological Methods*

*Antisera.*—These were obtained from birds bearing small tumours or those in which a tumour had regressed. Blood was taken at least 50 days after inoculation by which time the bird was expected to be a fully-immune carrier (Carr, 1944). Serum was heated to 53° for 2 hours, and then kept in the refrigerator until needed. The same batch of antiserum was used for all tests.

*Virus.*—This was a suspension purified according to the method of Bather (1953) in the case of the Rous I tumour, and a cell-free tumour extract, suitably diluted, for the other tumours. Its infectivity was titrated at the same time in young chicks according to the method of Carr and Harris (1951) with the corrections of Parker and Rivers (1936).

*Neutralisation.*—The standard method was used. Equal volumes of a stock dilution of virus, and a dilution of the antiserum were left for at least one hour to react, then 0·4 ml. of the mixture was injected into the breast or leg muscles of a group of 4–5 chicks, while a control of virus + saline was inoculated into the opposite site. The size of the resulting tumours is proportional to the amount of unneutralised virus.

At the end of 28 days, surviving birds were killed and the results recorded.

A typical result, showing partial neutralisation of III virus with Rous antiserum is shown in Table I.

The full neutralisation results are summarised in Table II.

No effective antiserum against II was obtained, as all inoculated birds grew a progressive tumour which killed them before 50 days had elapsed.

It is clear from this that III and Rous are closely related, but not identical. Since III antiserum completely neutralises Rous I, while the reverse neutralisation

TABLE I.—*Neutralisation of III Virus by Rous I Antiserum*

| Site | Inoculum | Tumour size Bird No. 1 | 2 | 3 | 4 |
|---|---|---|---|---|---|
| R. breast | III virus + Rous antiserum | — | — | — | — |
| L. breast | „ + saline | ++ | ++ | ++++ | ++++ |
| R. leg | „ + Rous antiserum × 1/5 | ± | ± | + | + |
| L. leg | „ + saline | ++ | ++ | ++++ | ++++ |
| | | 5 | 6 | 7 | 8 |
| R. breast | „ + Rous antiserum × 1/25 | +++ | +++ | +++ | +++ |
| L. breast | „ + saline | ++++ | ++++ | ++++ | ++++ |

Tumour size is indicated by the number of + signs.

TABLE II.—*Degree of Neutralisation of Viruses by Antisera*

| Virus | Infectivity | Antiserum | Final dilution 1/2 | 1/10 | 1/50 | |
|---|---|---|---|---|---|---|
| Rous | $10^{2}$ | Rous | C | C | C | |
| II | $10^{4.3}$ | „ | 0 | 0 | 0 | |
| III | $10^{3.3}$ | „ | C | p | t | |
| IV | $10^{1.3}$ | „ | 0 | 0 | 0 | |
| | | | 1/2 | 1/8 | 1/32 | 1/128 |
| Rous | $10^{3.3}$ | III | C | C | C | C |
| II | $10^{2}$ | „ | p | t | 0 | 0 |
| III | $10^{2.5}$ | „ | C | C | C | C |
| IV | $10^{2.3}$ | „ | p | t | 0 | 0 |
| Rous | $10^{3.3}$ | IV | 0 | 0 | 0 | 0 |
| II | $10^{2}$ | „ | 0 | 0 | 0 | 0 |
| III | $10^{2.5}$ | „ | 0 | 0 | 0 | 0 |
| IV | $10^{2.3}$ | „ | C | C | C | C |

Decreasing degrees of neutralisation are indicated as follows: C = complete, p = partial, t = trace, 0 = none.

is partial, it may be that III contains an antigen additional to those present in Rous I. The remaining viruses II and IV are distinct from both these and also from each other. The minor relationship of Rous I and II and III and IV, are of negligible importance, and there is always a doubt whether such weak reactions may not be due to naturally-acquired antibodies (Amies, 1937 ; Duran-Reynals, 1940*a*). This could only be decided, should the information be considered of importance, by repeated experiments using several antisera.

### DISCUSSION

These three tumours, derived from the first three specimens investigated, have yielded three quite distinct viruses. Such tumours are by no means rare, and the number of types of virus may therefore be great. One of these viruses has been identified as belonging to the Rous I group, the others are at present unclassified. That this cannot be done is mainly owing to the dearth of information in the literature. The last serious attempt at serological classification of the fowl neoplastic viruses was that of Andrewes in 1933. To-day, so far as is known, there is no place with a collection of known tumour viruses, let alone of antisera.

In view of the importance of classification as a preliminary to study, this position seems extraordinary. At present, it cannot even be decided whether the sarcoma viruses form many groups (the Rous I group and $MH_2$–$RF_4$ group of Andrewes (1933), etc.) or consist of a single group of immutable variants deriving from a single virus type, or are mutated varieties of either arising in the field. For this reason also their relationship (if any) with the erythroleukoses and lymphomatoses remain hypothetical, though by all other criteria they are obviously very similar to the former.

In the present series, the relationship of III and Rous I was suggested histologically from the beginning, and the serological confirmation of this was not unexpected. If histological criteria could be used, even in part, as a substitute for the laborious serological classification detailed here, this would be a notable preliminary simplification of the problems of distribution of viruses met in the field. In any case, this draws attention to the need for the development of a simpler method for making such identifications. The method used here, though sensitive and delicate as a research technique, is unsuitable for routine diagnostic work, even if classification could reduce the number of cross-tests needed to a few standard antisera. Unfortunately, the simple but precise methods based upon haemagglutination are not applicable to these or any other tumour viruses (Carr, unpublished).

No variation in any of these tumours has so far been noted, though this has sometimes been a marked feature of virus tumours after isolation, e.g. the reversion of the osteochondrosarcoma (Rous, Murphy and Tytler, 1912) to an undifferentiated type and the changes of Rous I itself (Rous, 1910). To what extent this is correlated with changes in the antigenic structure of the virus is unknown ; biochemical change there must be, of course. Such instability seems to be rather frequent, and indicates that care must be taken to preserve the original strain of virus as isolated.

### SUMMARY

Three new neoplastic visuses have been isolated from spontaneous myxomatous fibrosarcomata of the domestic fowl. One is closely related to Rous I serologically, though not identical with it, and the sarcomata it induced are similar in structure and behaviour. The other two are unrelated to this and each other.

### REFERENCES

AMIES, C. R.—(1937) *J. Path. Bact.*, **44**, 141.
ANDREWES, C. H.—(1933) *Ibid.*, **37**, 27.
BATHER, R.—(1953) *Brit. J. Cancer*, **7**, 492.
CAMPBELL, J. G.—(1945) *J. comp. Path.*, **55**, 308.
CARR, J. G.—(1944) *Brit. J. exp. Path.*, **25**, 56.
*Idem* AND HARRIS, R. J. C.—(1951) *Brit. J. Cancer*, **5**, 83.
DURAN-REYNALS, F.—(1940*a*) *Yale J. Biol. Med.*, **13**, 61.—(1940*b*) *Ibid.*, **13**, 77.
MURRAY, J. A. AND BEGG, A. M.—(1930) *Sci. Rep. Imp. Cancer Res. Fd., Lond.*, **9**, 1.
OLSON, C.—(1948) *Amer. J. vet. Res.*, **9**, 198.
*Idem* AND BULLIS, K. L.—(1942) *Bull. Mass. agric. Exp. Sta.*, No. **391**.
PARKER, R. F. AND RIVERS, T. M.—(1936) *J. exp. Med.*, **64**, 439.
ROUS, P.—(1910) *Ibid.*, **12**, 696.
*Idem*, MURPHY, J. B. AND TYTLER, W. H.—(1912) *J. Amer. med. Ass.*, **58**, 1840.